# Supplementary material for: The biosynthetic pathway to tetromadurin (SF2487/A80577), a polyether tetronate antibiotic
Source: PLoS One. 2020 Sep 14;15(9):e0239054. doi: 10.1371/journal.pone.0239054 (PMC7489565; doi:10.1371/journal.pone.0239054)
Supplement: Attachment — Submitted filename: Response to reviewers.pdf [file pone.0239054.s020.pdf]

Journal Requirements:

When submitting your revision, we need you to address these additional requirements.

1. Please ensure that your manuscript meets PLOS ONE's style requirements, including those for file naming. The PLOS ONE style templates can be found at https://journals.plos.org/plosone/s/file?id=wjVg/PLOSOne_formatting_sample_main_body.pdf andhttps://journals.plos.org/plosone/s/file?id=ba62/PLOSOne_formatting_sample_title_authors_affiliations.pdf

I believe we meet these requirements

2. We note you have included tables to which you do not refer in the text of your manuscript. Please ensure that you refer to Tables 1, 2 and 3 in your text; if accepted, production will need this reference to link the reader to the Tables.

The figures are now referred to in the text

We would like to thank the referees for their comments, which we feel have improved the manuscript. See below for our responses.

Reviewer #1: This manuscript describes the identification and characterization of the biosynthetic gene cluster for tetromadurin from Actinomadura verrucosispora. The authors present a thorough biosynthetic proposal based upon bioinformatic analysis of the gene cluster alongside several strategic gene inactivation experiments. Comparisons are drawn to the previously studied tetronasin and tetronomycin pathways.

The manuscript is well written with only a couple of very minor typographic errors and describes sound, high quality experimental research.
Publication is recommended after these minor changes.

Line 81-83: How were the necessary building blocks determined? Was this previously reported? Based on labelling experiments? Or is this a prediction?

A prediction by us. The building blocks were predicted from the structure of tetromadurin polyketide backbone. This has now been clarified in the text :

“*In contrast, on the basis of its structure we predicted that the 31-carbon polyketide skeleton of tetromadurin is synthesised using six malonyl-CoA, eight (2S)-methylmalonyl-CoA, and one uncommon (2R)-methoxymalonyl-ACP (acyl-carrier protein) unit (S1 Fig)***.”**

Line192: Add information about the MS parameters used for HPLC-MS analysis.

The following sentence has been added in the methods:

"The mass spectrometer was operated in positive electrospray ionisation mode set to full scan (from m/z 200-2000)."

Line 213-214: Include information in methods section about how the Actinomadura verrucosispora was sequenced. Include gDNA isolation and sequencing preparation steps as well as genome assembly information. If this was carried out by a service, state which company/lab

The method for genomic DNA preparation has now been added to the methods section

"For the preparation of genomic DNA (gDNA), 250-500 µL of mycelia was centrifuged in a 1.5 mL microcentrifuge tube to pellet the cells and the supernatant was discarded. The cell pellet was resuspended in 500 µL of SET buffer (20 mM TrisCl, 75 mM NaCl, 75 mM EDTA, pH 7.2) containing 10 µL of lysozyme solution (50 mg/mL). After incubation at 37 °C for 1 h, 60 µL of 10% SDS (w/v) and 10 µL of proteinase K (20 mg/mL) were added and the tube was incubated at 55 °C for an additional 2 h. The sample was then mixed with 300 µL of 5 M NaCl and 500 µL of chloroform and centrifuged at 2200 x g for 15 min. The aqueous upper layer was transferred to a fresh microcentrifuge tube using a T1000 pipette tip with the end cut off to avoid shearing the gDNA. To precipitate the gDNA, 0.6x volumes of isopropanol was added followed by gentle mixing. The precipitated gDNA was washed twice in 70% ethanol before being air dried for 5-10 min. The gDNA pellet was dissolved in 100-200 µL of distilled water."

…

"Include gDNA isolation and sequencing preparation steps as well as genome assembly information"

The method for genomic DNA sequencing and assembly (including the genbank accession number) has also been added:

"Nextera shotgun and Nextera mate-pair libraries were constructed from high molecular weight genomic DNA isolated from *Actinomadura verrucosospora* NRRL-B18236 Sequencing was carried out on an Illumina MiSeq platform using the Illumina V2 500 cycles kit in 2 × 250 bp mode. Reads processed using a custom adapter trimming tool(fastq_miseq_trimmer). Read pairs were then preassembled using FLASH v1.2.11 (https://ccb.jhu.edu/software/FLASH). For de novo assembly we have used newbler v3.0. Several assemblies were carried out using either all or subsets of the input dataset, and the best assembly was selected using a score calculated from scaffold N50, edge and total number of contigs. The best assembly had been polished using Pilon. ORFs were predicted ab initio using a customised version of the FGENESB pipeline V2.0 (2008) [www.softberry.com] and blasted against filtered NCBI NR and KEGG datasets.

Customised linguistic analysis was used for transfer of gene annotations. Annotation results were saved in EMBL format and manually curated in Artemis (http://www.sanger.ac.uk/science/tools/artemis). The revised genome sequence has been deposited in GenBank under accession number CP053892."

Figures 4/5: would benefit from using colour to highlight chemical changes in the structures. Also hard to read the writing inside circles in Fig 4, remove shading in circles.

Good idea. Colour has now been added to these figures to highlight the chemical changes occurring. The shading in the circles representing PKS domains has also been removed.

Line 282-285: These two sentences feel out of place a confusing. Either remove entirely or rephrase and move to after the description of the PKS genes that are present.

These sentences have been removed, as the absence of the PKS "D" genes is apparent from Figure 2.

Line 300-303: The use of the term "predicted" to describe both of the structures in Fig 6 caption is confusing and inconsistent with the statement in line 292-294 in the text. Please clarify language.

The main text has been updated to clarify that both structures have been predicted, just through different means.

"The linear polyketide predicted by ordering the PKS enzymes MadAI-MadAVII (**13**) (i.e, MadAI initiates tetromadurin biosynthesis and performs two extension cycles before transferring the nascent polyketide chain to downstream to MadAII etc···) is in near perfect agreement to the linear polyketide we predicted from retrobiosyntheic analysis of tetromadurin itself (**14**) (**Fig 6**). The placement of the C5-C6, C10-C11, C12 and C13 double bonds in **14** was guided by the recently isolated partially cyclised tetronasin intermediate [7]."

Line 421: Change "(Fig8c)" to "(Fig 9c)"

Done

Line 500-502: Can you really use this argument if the complementation worked for Mad10 using the same vector?

The reason for the failed complementation is unclear, especially since the deletion was made in the coding frame of the gene to rule our polar effects on adjacent genes. Integration and expression from phage sites can be difficult to predict (especially if multiple sites are present in a genome), so we still think some expression issue is the most likely explanation.

These sentences have been updated:

"The failed mad31 complementation, paired with the mad10 complementation only restoring tetromadurin production in A. verrucosispora ∆mad10 to 28% wild type levels, suggest expression issues of the streptomycete-optimised vector pIB139 within an Actinomadura strain."

Line 533: Change "Fig 7" to "Fig 8"

Done

Line 756: Change "oolyether" to "polyether"

Done

Reviewer #2: Little et al identified the biosynthetic gene cluster involved in polyether compound tetromadurin biosynthesis in Actinomadura verrucosispora. They did bioinformatic analysis and performed gene deletions (cyclase genes mad10 and mad31) to confirm the involvement of this cluster in tetromadurin biosynthesis. The overall writing is clear. The following issues are suggested to be addressed:

The biggest issue is that the novel tetromadurin analogue produced by Δmad10 was not purified and identified by NMR analysis.

It is true that the tetromadurin intermediate produced by the Δmad10 mutant (T-17) was not isolated for NMR analysis. However, given the recently in-depth structural characterisation we performed on the tetronasin intermediate from *S. longisporoflavus* Δtsn11 (called 6 in the original submission of this manuscript, now 17), we believe the mass spectrometry data is sufficient – particularly the observation that T-17 contains an additional, labile, hydroxyl group (suggesting the tetrahydropyran ring is unformed). Ultimately the aim of this paper is present the biosynthetic gene cluster of tetromadurin and use the available experimental data to propose a reasonable pathway for its biosynthesis.

Fig 4, 5: please combine these two figures. For Fig 4, I would like to suggest the authors to use differentcolors to indicate the specificities of the ATs.

During the preparation of this manuscript combining these two figures together was attempted. However, due to the size of the images and the level of detail, it was not possible to create a single figure that complied with the figure requirements of PLoS (the figure was either too big, or had text/chemical bonds too small to be interpretable).

However, colours have now been added to indicate the AT domain specificities.

Fig 6: It is more appropriate to remove Fig 6 to supporting information as this product is predicted via bioinformatics analysis.

While these structures are predictions, they are important for following the arguments presented in the manuscript. This figure is also useful to have in the main text during the discussion of the apparently inactive KR domain of module 12. As such, we would prefer to have it remain in the main text.

In the section of "Genes for (2R)-methoxymalonyl-ACP biosynthesis", line 381-382, tautomycin and oxazolomycin are suggested to be referred as well.

The papers describing the biosynthesis of these compounds have now been referenced. See references 33 and 34.

Fig 9: It is more appropriate to remove Fig 9 to supporting information.

This figure presents the structure of the tetronasin intermediate 17 (called 6 in the first submission) isolated from *S. longisporoflavus* Δtsn11. The structure of 17 was critical in guiding our predictions for the structure of T-17 from *A. verrucosospora* Δmad10 , so we believe should be readily accessible in the main text to help the reader.

S13 Fig: more PyrE3 homologues are suggested to be subjected to phylogenetic analysis.

When running a BLAST search on PyrE3 the majority of hits are for FAD-dependent monooxygenase enzymes. It is still difficult to determine with certainty if a PyrE3 homologue is involved in a spirotetronate pathway or is a real monooxygenase. The homologues included are from known spirotetronate biosynthetic pathways, of which only a few are currently known.

Line 987: "Fig Alignment of" should be "S14 Fig Alignment of" The accession numbers of protein sequences for alignment are suggested to be added.

The accession numbers have now been added.

Please number all the compounds in the manuscript.

All compounds have now been given an identifying number.
